# Pharmaceutical policies and regulations of oral antiviral drugs for treatment of hepatitis C in Egypt—case study

**DOI:** 10.1186/s40545-021-00389-6

**Published:** 2021-12-16

**Authors:** Mahmoud H. Teaima, Adi Al-Nuseirat, Dalia Abouhussein, Osama A. Badary, Mohamed A. El-Nabarawi

**Affiliations:** 1 Department of Pharmaceutics and Industrial Pharmacy, Faculty of Pharmacy, Cairo University, Cairo, Egypt; 2 Access to Medicines and Health Technologies Unit, World Health Organization Office for the Eastern Mediterranean Region, Cairo, Egypt; 3 Pharmaceutics Department, Egyptian Drug Authority (EDA), Cairo, Egypt; 4 Department of Clinical Pharmacy Practice, Faculty of Pharmacy, The British University in Egypt, Cairo, Egypt

**Keywords:** Pharmaceutical regulations, Direct-acting antivirals, Hepatitis C virus

## Abstract

**Background:**

There are limited studies on the role of efficient regulatory mechanisms in facilitating greater access to Hepatitis C virus (HCV) treatment. Evidence to support the importance of effective pharmaceutical policies and regulations in improving access to oral viral drugs towards the elimination of HCV is needed. This study aims to explore the adequacy of the implemented pharmaceutical policies and regulations in Egypt and their role to improve the availability and affordability of direct-acting antivirals (DAAs) to achieve universal access to the treatment of HCV.

**Methods:**

The study adopts a qualitative methodology using desk review of regulatory and legislative information, literature review, and semi-structured interviews with key experts from the concerned governmental regulatory agencies, pharmaceutical industries, academic organizations, professional associations, civil society organizations, and clinicians who are working in researching treatments for hepatitis C.

**Findings:**

The common DAAs available in the market are Daclatasvir, Sofosbuvir, and Sofosbuvir-based direct-acting antiviral combinations. Fast-track medicines registration pathway for marketing authorization of DAAs is used to reduce market access time frames. The pricing policies are supplemented using price negotiation to set up affordable prices that led to a reasonable price for DAAs. Using Trade-Related Aspects of Intellectual Property Rights (TRIPs) flexibility and local production of quality generics DAAs at lower prices. In addition, political will and collaboration between the government, civil society, and pharmaceutical companies improved patients' access to affordable DAAs and succeeding hepatitis C treatment in Egypt.

**Conclusions:**

The study findings indicated that the implemented pharmaceutical policies and regulations have an immense role in enhancing access to medicines towards the elimination of hepatitis C in Egypt.

**Supplementary Information:**

The online version contains supplementary material available at 10.1186/s40545-021-00389-6.

## Introduction

Hepatitis C virus (HCV) is the major cause of chronic liver disease, hepatic cirrhosis, and hepatocellular carcinoma, as well as the most common reason for liver transplantation in many countries [1]. The World Health Organization (WHO) estimated that 71 million people have chronic hepatitis C infection, and approximately 400,000 die from the disease each year, mainly from cirrhosis and hepatocellular carcinoma [2]. In Egypt, over 10% of the population is predicted to be HCV positive, of which 94% are infected with genotype 4 [3, 4]. By 2015 hepatitis C accounted for 40,000 deaths per year in Egypt, accounting for 7.6% of all deaths in the country and a 1.5% depression of national Gross Domestic Product (GDP) growth [5].

Until recently, the standard care for HCV infection is comprised of injectable peg-interferon used in combination with ribavirin (RBV) [6]. This treatment has several disadvantages: too long, too expensive, often associated with strong side effects, and a low cure rate that does not exceed 50% depending on the genotype of the virus and the stage of liver damage [7]. A new class of oral antiviral drugs called direct-acting antivirals (DAAs) has, for the first time, provided an opportunity for widespread scale-up of curative treatment. DAAs oral medications have been proven to be safe and effective in clearing the HCV from the body within 12 weeks of treatment. Moreover, the most recent products have a high sustained virologic response (SVR) in all patient subgroups regardless of the genotypes, age, gender, race, and levels of the liver enzyme [8, 9].

Every single disease management approach demands access to medicines for prevention and treatment. Elimination of disease is not possible if people don’t have reliable and sustainable access to quality-assured medicines and health products. The national regulatory authority (NRA) is the institution that promotes and protects public health by assuring the quality, safety, and efficacy of medical products as well as ensuring the relevance and accuracy of product information. The official drug regulatory body in Egypt has been operated under the patronage of the MOHP till recently. In August 2019, Egypt issued Law No. 151 of 2019 [10] to establish the Egyptian Drug Authority (EDA) along with the Egyptian Authority for unified drug procurement. According to the law, the EDA is a public service body affiliated to the prime minister and replaces the minister of health and population in the specialties stipulated in Law No. 127 of 1955 [11] regarding practicing the pharmacy profession, regulating the registration, as well as handling and controlling medical preparations and supplies. In January 2020 the president of Egypt issued the presidential decree 18/2020 [12] to give the EDA a more autonomous status. Based on the recent law, the EDA will be responsible for developing and implementing policies and regulations to ensure access to safe and effective pharmaceutical products, and will comprise of three sub-organizations, including the Central Administration of Pharmaceutical Affairs (CAPA), which is responsible for registering pharmaceutical products, pricing of medicines, issuing licenses for the establishment of pharmaceutical entities, inspecting the pharmaceutical facilities, licensing the importation and exportation of pharmaceutical products, monitoring post-marketing surveillance, and detecting and assessing adverse drug reaction. The National Organization for Drug Control and Research (NODCAR), which oversees lab testing of pharmaceutical products, medical devices, in-vitro diagnostics, veterinary products, and cosmetics to evaluate their compliance with the required standards and confirm their safety and effectiveness. And the National Organization For research and Control of Biologicals (NORCB), which is responsible for monitoring the safety, quality, and efficacy of the biologicals.

In May 2016, a historic commitment to eliminate viral hepatitis by 2030 was made by 194 Member States, at the 69th World Health Assembly, governments adopted the “Global Health Sector Strategy on Viral Hepatitis, 2016–2021” [13]. The strategy emphasizes the important role of universal health coverage and sets targets that align with those of the Sustainable Development Goals (SDGs), it has the vision to eliminate viral hepatitis as a public health issue. This is enclosed in the global targets to reduce new viral hepatitis infections by 90% and decrease deaths because of viral hepatitis by 65% by 2030. Actions to be taken by countries and the WHO Secretariat to reach these targets are outlined in the strategy [14]. However, to meet these goals, the bottom line of the challenge ahead is how to provide access to such high-cost medicines that used to treat HCV infection. This study describes the current pharmaceutical regulations of the new oral antiviral drugs to treat hepatitis C in Egypt and examines its implication and contribution to eliminate HCV. As there are limited studies on the role of efficient regulatory mechanisms in facilitating greater access to HCV treatment, this study will provide evidence to support the importance of effective pharmaceutical regulations in improving access to oral viral drugs and the elimination of HCV. The findings of the study can be used by national and universal stakeholders to improve the pharmaceutical regulations and regulatory systems of medicines used to treat hepatitis C.

## Methodology

The objective of this study is to answer the question of “whether the current national pharmaceutical policies and regulations including legal, administrative and supply of oral antiviral drugs, specially DAAs improves access to hepatitis C treatment in Egypt”. To answer this question, we examined four key domains:

1. The current regulatory requirements and procedures by the Egyptian drug authorities involved in granting market authorization of DAAs in Egypt.
2. The present pricing policy and mechanisms for pricing medicines and DAAs, because we want to examine if the pricing policy in Egypt is effective in managing the prices of DAAs and leads to a convenient and affordable price, accordingly we compared the prices of selected DAAs in Egypt to the prices in other neighboring countries. These countries have been selected, because their prices are publicly available on the government’s websites.
3. The national intellectual property and patent protection legislation in Egypt and its relation to access to DAAs products.
4. Local production of safe, effective, and affordable generic DAAs in Egypt.

### Design

This research adopted a qualitative approach to cover the whole of Egypt that includes a desk review of regulatory and legislative information, literature review, and semi-structured interviews.

### Data collection procedure

For the purpose of this study, data was collected from several resources. First is the desk review of regulatory and legislative information was collected from government reports and guidelines on regulating DAAs products in Egypt including data from the relevant websites of the stakeholder organizations, such as the Egyptian Drug Authority (EDA), Egyptian Patent Office (EGPO), Ministry of Health and Population of Egypt (MOHP), Ministry of Trade and Industry, Ministry of Finance, Egyptian Pharmacist Syndicate, The Egyptian Society of Hepatology, Gastroenterology and Infectious Diseases (ESHGID), and Pharmaceutical Egyptian Association. Data on the registration status of DAAs in Europe and the United States of America (USA) was collected from the web pages of the European Medicines Agency (EMA) and the United States Food and Drug Administration (FDA). Data on prices of registered DAAs in Jordan, Lebanon, Morocco, Saudi Arabia, and the United Arab Emirates (UAE) was collected from the websites of the medicine’s regulatory authorities to be compared with the prices in Egypt. As these data are considered the key source of data for this study, to assure the data quality, all the internet and printed documents were collected from governmental and well-respected organizations’ websites. Second, articles referring to the regulation of medicines were reviewed, however, as expected the data available from academic journals was limited as this area of study is under-researched. Third, interviews with multi-stakeholders and key experts in the area of marketing authorization, pricing, patency, and local production of DAAs medicines, were conducted to obtain data on the role of the government in promoting investment in the development and production of such crucial products. The findings were then contrasted with the data obtained from the desk and literature reviews. This was an important step, since key informants can provide rich, sensitive, actual, and up-to-date information that is otherwise not available from documents’ review [15].

### Interviews and participants inclusion criteria

Homogeneous sampling, a type of purposive sampling method, was used to identify relevant key informants for the interviews [16]. Once the initial interviewee list was developed and data collection had begun, the snowball technique was used to add additional potential interviewees from a referral. The interviews continued until we reached the point of data saturation. A total of 31 interviews were conducted with key experts from the concerned governmental regulatory agencies, pharmaceutical industries, academic organizations, professional associations, special interest groups, and clinicians who are working in researching treatments for hepatitis C in Egypt. The selection was based on the level of expertise and understanding of the subject of research [17]. The potential to provide valuable data that may not be in the public domain. The number of key informants per section was designed based on the below criteria:

1. At least 15 years of knowledge in medicines regulation and 2–5 years of which in the regulation of DAAs in Egypt.
2. At least 15 years of experience in the pharmaceutical industry with at least 2–5 years in DAAs research and development, production, marketing, safety, and efficacy.
3. At least 10 years of experience in policymaking position in medicines regulation including procurement and pricing policies.
4. At least 10 years of experience in clinical research/practice in the treatment of hepatitis C and liver diseases in Egypt.
5. At least 7 years of experience in patent examination and negotiation of intellectual property rights agreements at national and international levels.

### Interview process

Introductory email messages were sent to the interviewees for introducing the Principal Investigator (PI) and explaining, in brief, the objectives of the study and inviting them to be part of the research project. The interviewees were reassured that their names would be kept confidential. All interviews were pre-appointed and lasted between 45 min to 1 h. Due to the COVID-19 pandemic, all interviews were executed via telephone calls and using video conferencing technology (Additional file 1).

Semi-structured interviews were conducted when needed to obtain valid and accurate data directly from stakeholders and specialists. The themes of the interviews and some pre-prepared open-ended questions were drawn up before each interview, but the actual interview and its focus varied according to the expert’s position, experience, and emergent information. Information and material from previous interviews were utilized in subsequent interviews. Furthermore, some interviewees were requested to provide documents to validate positive responses. As mentioned previously, the main source of data for this study was document data. The data obtained from the interviews were, therefore, used to verify the findings from the document analysis. When there was no document data available, but the findings from the interviews were considered relevant, a citation noting that the data came from an interview is included.

## Results

The EDA is responsible for evaluating marketing authorization applications. Pharmacies are prohibited from selling medicines that are not registered through a ministerial decree that provides for the requirement to register medicines before being allowed to circulate in the Egyptian market. It further provides powers to the regulatory authority to issue, suspend or withdraw marketing authorization for medicines.

There are several scientific committees involved in registration to assure that the applications submitted for registration are fully assessed for efficacy, safety, quality, accuracy, and completeness of product information. The EDA website is very informative and includes lots of resource documents that describe activities, procedures, decrees, and decisions; however, it needs to be more structured so that the data can be easily found. Nevertheless, the EDA announced a new website that will encompass all EDA activities but is still under construction.

Due to the importance of DAAs, Egypt has exempted the DAAs from the condition that they should be registered and distributed in the country of origin or the reference country for at least 1 year. Fast track registration of DAAs generics have been granted to interested companies, and the government promotes and facilitates local production of HCV treatment in order not to allow for market monopolization by multinational companies, they also allow the manufacturers to register an unlimited number of them.

It has been noticed that within less than 3 months of registration of DAAs innovators, EDA has approved marketing authorization of the low-price generics. Although Egypt succeeded in registering the DAAs (innovators and generics) in short periods, recommendations were suggested during the interview to enhance management practices and improve governance and transparency through improving online registration information system, digital archiving of administrative–technical documents, and public dissemination of guidelines, procedures and decision-making process related to the regulation of medicines. As for January 2021, there are 71 DAAs registered in Egypt, including four innovator brand medicines and 67 generics.

The market price of innovator brand is determined according to the least selling price, where the product is marketed in a reference list of 36 countries [18], it includes Algeria, Argentina, Austria, Bahrain, Belgium, Canada, Cyprus, Denmark, Finland, France, Germany, Greece, Hungary, India, Iran, Ireland, Italy, Japan, Jordan, Kuwait, Lebanon, Morocco, Netherlands, Norway, Oman, Philippines, Poland, Portugal, Saudi Arabia, Spain, Sudan, Sweden, Switzerland, Turkey, United Arab Emirates, and the United Kingdom. In case the product is marketed in less than five countries, pricing should be done either following a comparative study between the product in question and its therapeutic alternatives or as per the least of the prices in those five countries. The applicant should submit to EDA a list of market prices of the product in countries, where it is registered, including all discounts, if any.

As for generics whether imported or locally produced, they are subject to a markdown percentage from the branded product price of 35% for the first five generic products who apply for pricing, and 40% for any other generic products which follow in the “box”. Imported “high technology” generic products are subject to a 30% markdown of their branded version in case the product was manufactured in a reference country (ICH country) and 35% if manufactured in a non-reference country [19].

Profit margins for pharmacists and distributors vary based on the pharmaceutical product categories, such as medicines in the essential medicines list, imported products, and locally produced products. Tariffs, taxes, and customs differ from one product to another according to importance. In Egypt tariffs, customs and taxes are applied to all medicines except medicines for chronic diseases. Since the treatment of HCV in Egypt has become one of the topmost national priorities, the DAAs whether locally produced or imported and their active substances involved in their manufacturing, are exempted from taxes and customs.

According to decree 499/2012, pricing revisions are done every 5 years; however, there are cases when revisions are necessitated: (1) currency exchange fluctuations of average 15% up or down in 1 year; (2) when a company requests price revision of its products, not exceeding 5% of its products per year. In cases when following the pricing legislation in force would cause the medicine to be priced at a relatively high price, the committee would start a process of price negotiations with the producing company to set a lower price. As the prices of brand DAAs are considered very expensive and unaffordable to the Egyptians when applying the national pricing legislation, Egypt started the process of negotiation with the DAAs manufacturing companies. Egypt has managed to negotiate a price reduction of this expensive treatment and succeeded in getting the supply of some DAAs at a 99% discount to the U.S. price. The government also encouraged local production and registration of DAAs generics at prices less than 20% of branded DAAs. The prices of DAAs in Egypt are also considered affordable if it compares with the prices of neighboring countries. It is worth mentioning that the price of DAAs in the public sector may reach less by 50% of the price in the private sector.

The DAAs didn’t get patent protection from the EPGO due to various reasons. Some patent application has been rejected by the EGPO, because it does not fulfil the patentability criteria of novelty and inventiveness, in accordance with the Egyptian law on intellectual property rights. Others didn’t fill the patent applications. As the DAAs didn’t obtain patent protection, this granted the local pharmaceutical manufacturers the authority to produce generic versions of DAAs locally without IP-related restrictions.

Egypt has the biggest pharmaceutical industry base in the middle east that was founded since 1939 with a presence of around 120 pharmaceutical companies, of which fewer than ten are multinationals with local production bases. Pharmaceutical production in Egypt is mainly based on manufacturing finished dosage forms, with some local production of active pharmaceutical ingredients (APIs).

Several studies showed that the locally produced generic direct-acting antivirals (DAAs) are just as effective and safe as their branded equivalents for the treatment of chronic hepatitis C virus (HCV) infection [20, 21]. Key informants’ clinicians indicated that generic DAAs used in treating patients demonstrated equal potency, safety, and tolerability compared to original brands. To promote local pharmaceutical production, MOHP has sourced medicines for the hepatitis C treatment campaign exclusively from domestic generic manufacturers. Innovator companies importing or manufacturing in Egypt have been lowering their prices to be competitive in the private market, but their volume share will probably remain limited.

## Discussion

This is the first study that pursues to explore the competence of the implemented pharmaceutical policies and regulations in Egypt and their role to improve the availability and affordability of direct-acting antivirals (DAAs) to achieve universal access to the treatment of HCV. The study investigates the following fields: regulatory structure, marketing authorization procedure, pricing policy, Intellectual property and patent protection, and local production of DAAs.

### Regulatory structure

NRAs are the doorkeepers of the supply chain of medical products, and they have a mandate to ensure the quality, safety, and efficacy of medicines and health products. They work within a legal framework and set of regulatory functions spanning the medical product lifecycle, from clinical trial oversight, product marketing authorization and registration, licensing pharmaceutical establishments, Inspection of manufacturers, importers, wholesalers, and dispensers of medicines, testing products, controlling of importation, exportation, and promotion of medicines, post-marketing surveillance, and vigilance activities [22–24].

The Egyptian Drug Authority (EDA) has been established in 2019 as an independent national competent authority to ensure safety and quality of medicines and pharmaceuticals. The law governing the regulation of pharmaceuticals in Egypt is the Pharmacy Act of 1955. The law regulates the registration of pharmacists, control of pharmaceutical institutions, such as community pharmacies, pharmaceutical products manufacturers, and drugs store. In addition, the law provides for registration of medicines, control of pharmaceuticals imports and exports, and manage medicines prices.

### Marketing authorization procedure

Registration of medicines (marketing authorization or product licensing) forms one element of the medicine regulatory control system. It is a procedure of release medicines for marketing after it has undergone a process of evaluating their safety, efficacy, quality, and the appropriateness of the product information. Article 59 of Law 127 for 1955 [11] prohibited pharmacies from selling medicines that are not registered. Medicine either locally manufactured or imported should be registered in the EDA and given a registration number before it can be traded or given to people.

The authorization process is regulated by ministerial decree 425 of the year 2015 [25]. The registration guideline explains the procedure of registration to evaluate the safety, efficacy, and quality of the product, and the appropriateness of the product information. After the product has been assessed and confirmed its safety and efficacy, the EDA will issue the final approval for the applicant valid for 10 years from the date of the issuance of the initial approval. There is no pre‐defined registration timeline using this ministerial decree. Therefore, the time needed to complete registration can vary from 6 months for a priority life‐saving product to 2 years for the other products; some key informants said it may extend to more than 4 years.

The HCV-DAAs are categorized into three classes depending on their function: NS3/4A protease inhibitors, NS5A inhibitors, and NS5B polymerase inhibitors [26]. The NS3/4A protease inhibitors have become less popular and are gradually disappearing from the market as more effective HCV-DAAs with fewer side effects became available. The NS5A inhibitors are effective, although they do not have a high genetic barrier to resistance, for this reason, NS5A inhibitors are combined with other DAAs that have higher resistance barriers, such as Ledipasvir–Sofosbuvir; such treatments have cure rates approaching 99% [27, 28]. The NS5B polymerase inhibitors include Beclabuvir, Dasabuvir, Filibuvir, Setrobuvir, and Sofosbuvir.

Sofosbuvir (Sovaldi®) manufactured by Gilead Sciences, Inc company was the first product that could be orally administered without the use of PEG-IFN, which has been approved through the accelerated pathway by the US FDA in December 2013 [29], it was also the first HCV DAAs approved in Egypt in July 2014. In October 2014 CAPA approved the first generic of Sofosbuvir. As for January 2021, there are 37 generics of Sofosbuvir in Egypt, of which all are locally manufactured.

Daclatasvir (Daklinza®) is the first and only NS5A inhibitor that was approved as a separate entity by US FDA in July 2015 [30], it has been registered in Egypt in August 2015, the first generic of it was approved by CAPA in October 2015, as for January 2021, there are 15 generics of Daclatasvir in the Egyptian market. The first multi-class combination drug, a combination product of ledipasvir & sofosbuvir (Harvoni®), has been registered in US FDA in October 2014 [31]. It showed an SVR of close to 99% after 12 weeks of treatment [32]. Its response rate was equally the same in most patient subgroups regardless of age, sex, race, liver enzyme levels, genotypes, and pre-existing antiviral resistance variants. Harvoni® has been approved in Egypt in October 2015 and after a few days, the first generic was approved by CAPA. Currently, there are 15 generics of this combination in Egypt.

As for January 2021, there are two single DAAs registered in the Egyptian market, which are: Daclatasvir and Sofosbuvir, after the discontinuation of Simeprevir, Telaprevir. There are also four multi-class combinations of DAAs available in the Egyptian market, which are: Sofosbuvir plus Ledipasvir; Sofosbuvir + Ledipasvir + Glecaprevir; Sofosbuvir plus Velpatasvir; and Elbasvir plus grazoprevir.

### Pricing policy and pricing mechanisms

Historically, since 1950 a strict compulsory pricing policy has been in place, intending to make medicines affordable to the lowest socioeconomic segments of the population, later formulated into legislation that was based on cost-plus and mark-up regulation. In 2009, external reference pricing was introduced and later combined with mark-up regulation in more recent legislation in 2012 [18]. The prices of pharmaceuticals are determined during the registration process through a pricing committee and based on a ministerial decree 499/2012 [19] that has come into force since July 2012. It combines External Reference Price (ERP) with markup regulation, detailing profit margins for pharmacists and distributors.

The price of Sofosbuvir (Sovaldi®) in the USA was announced to be US$ 84,000 per 12-week course, or US$ 28,000 per box [33]. This price is being an obvious impediment to access to treatment in low- and middle-income countries, where HCV is most prevalent. According to the pricing system in Egypt, a bottle of 28 tablets of Sovaldi® should have been priced at 103,699 LE (≈ 14,814 US$ at that time in 2015). Gilead had Egypt as the first country on their list for priority registration, which is based on prevalence, disease awareness, and regulatory pathway and timelines. Negotiations between the company and the Egyptian government, represented by the National Committee for the Control of Viral Hepatitis, resulted in what was perceived to be a good deal: US$ 300 per box of Sovaldi (28 tabs) as a 99% discount of the original Sovaldi price in the USA. This is the price to be paid by the government for the supply of Sovaldi to be provided for patients on government treatment registers. In other words, patients will not benefit from this price should they privately pay for their treatment. That same product was registered for the private market at EGP 14,900 per box (US$ 2130). This means that purchasing one box of Sovaldi® privately outside the national treatment programme would cost a patient what the government pays for a 12-week treatment course [34]. Currently, the price of Sovaldi® in the private market has been decreased to 4840 EGP (≈ US$ 308) per box. Allowing for local generic competition drove the price down further to 900 EGP (≈ US$ 57). This means that the treatment of hepatitis C by Sofosbuvir in Egypt ranges from US$ 170 using generics to US$ 924 using innovator, compared to US$ 84,000 in the USA.

EDA also reviewed the price of Daclatasvir (Daklinza®), which originally entered the USA market at US$ 63,000 for a 12-week course [35] and decided to be EGP 8000 (US$ 509) per box, for generics and because of the competitions, the price is EGP 120 (US$ 8). The initially published price in the US for the innovator Ledipasvir plus Sofosbuvir (Harvoni®) for 12-week courses of treatment was US$ 94,500 [36]. In Egypt, the price per bottle of Harvoni® for a 1-month supply is EGP 5500 (US$ 347). The price of generics is around EGP 1100 (US$ 70). Table 1 shows the prices of selected registered DAAs in Egypt.

**Table 1:** Prices of selected registered DAAs in Egypt

Generic name	Strength & dosage form	Average price of one box (28 tablet) in EGP*	Price 1 box (28 tablet) in USD
Daclatasvir—Innovator	60 mg tablet	EGP 8000	USD 509
Daclatasvir—Generics	60 mg tablet	EGP 120	USD 8
Sofosbuvir—Innovator	400 mg tablet	EGP 4840	USD 308
Sofosbuvir—Generics	400 mg tablet	EGP 900	USD 57
Sofosbuvir, Ledipasvir—Innovator	(400, 90) mg tablet	EGP 5500	USD 350
Sofosbuvir, Ledipasvir—Generics	(400, 90) mg tablet	EGP 1100	USD 70
Sofosbuvir, Velpatasvir—Generics	(400, 100) mg tablet	EGP 2000	USD 127

*Egyptian Pound equals 0.0636 United States Dollar on 20 January 2021

To illustrate more the prices of DAAs in Egypt, we selected five Arab countries based on the accessibility and availability of data on the prices of registered DAAs at their NRA’s websites, to compare it with prices of DAAs in Egypt. The selected countries are Jordan, Lebanon, Morocco, Saudi Arabia, and the United Arab Emirates. Morocco is a Lower-middle-income country such as Egypt, Jordan and Lebanon are Upper-middle-income, while Saudi Arabia and the United Arab Emirates are high-income countries [37]. Table 2 compares the prices of treatment of hepatitis C using different DAAs innovators between Egypt and five Arab countries (Jordan, Lebanon, Morocco, Saudi Arabia, and the United Arab Emirates) [38–43]. The prices have been collected from the MOH or NRA website in these countries, the prices converted from national currencies to US dollars using average exchange rates at the time data was collected. The data confirmed that the prices of DAAs in Egypt are the lowest in the region.

**Table 2:** Prices of Harvoni®, Epclusa®, and Daklinza® in Egypt, Jordan, Lebanon, Morocco, Saudi Arabia, and the United Arab Emirates

Name of medicine	Price in Egypt for a 12 weeks	Price in Jordan for a 12 weeks	Price in Lebanon for a 12 weeks	Price in Morocco for a 12 weeks	Price in Saudi Arabia for a 12 weeks	Price in UAE for a 12 weeks
Harvoni® (Sofosbuvir + Ledipasvir)	1050 USD	67,000 USD	49,000 USD	1600 USD	66,450 USD	74,000 USD
Epclusa® (Sofosbuvir + Velpatasvir)	*432 USD	38,840 USD	34,380 USD	1900 USD	37,700 USD	69,250 USD
Daklinza® (Daclatasvir)	1527 USD	35,470 USD	NA	NA	33,252 USD	NA

*Epclusa® is not registered in Egypt, the generic of it is produced by a local company

The Egyptian Authority for Unified Procurement (UPA) was established in 2020 as a public economic authority to report directly to the Prime Minister based on law No. 151 of 2019 [10]. UPA will be the exclusive authority to carry out purchase transactions of pharmaceutical products and medical equipment on behalf of all governmental and public entities in Egypt. According to the law, the governmental and public entity will be prohibited from making any direct purchase of pharmaceutical products or medical equipment except in case of emergency and after obtaining the approval of the Cabinet of Ministers. The Egyptian government usually procures medicines for use within the public sector at relatively low prices compared to private sector prices. Procurement of pharmaceutical products in the public sector follows a Tender Drug List (TDL) system [44, 45].

### Intellectual property and patent protection

Egypt was a signatory of the TRIPS agreement in 1995 and joined the World Trade Organization in 1996. By signing the agreement, Egypt was committed to bringing its Intellectual Property Rights (IPR) legislation into compliance with TRIPS, which was achieved in 2002 with the issuance of Law No. 82 on protecting IPR, and which has entered into force since 1 January 2005 [46].

The EGPO is the entity responsible for receiving, assessing, and making decisions on patent applications filed in Egypt. It is accredited by the World Intellectual Property Organization (WIPO) as a regional searching authority and plays a key role in technology transfer, IPR protection, and the creation of enabling environment for STI‐based business and investment. In September 2009, during the international meetings of the General Assembly of the WIPO, the EGPO was accredited as an International Searching Authority and International Preliminary Examining Authority (ISA/IPEA) under the Patent Cooperation Treaty (PCT), and it is currently authorized to accept patents applications from all over the world [47]. EGPO monthly publishing the official patent gazette which includes filed, accepted application, granted patents done applications and patents. Patents in the latest law are granted for 20 years; however, the law has several articles called TRIPS flexibilities which safeguard the protection of public health and public interest and prevention of the abuse in exclusive exploitation of the patent.

Patents for pharmaceuticals form an important part of the TRIPS agreement, giving patent holders monopoly rights over their inventions, as they provide the inventor with the legal means to prevent others from “manufacturing, offering for sale, selling, or importing” any new invention, whether it involves a product or a process. Consequently, patents lead to an increase in the price of essential medicines to become unaffordable to billions of people in the developing world [48]. However, new medicines arriving on the market are not all protected by patents systematically [49]. Examples where patents do not apply include:

- When an innovator company may have never applied for patent protection.
- A patent application may have been rejected by the patent office for non-compliance with the country’s patentability criteria.
- And, under the TRIPS agreement, governments have the right to suspend patent protection on a key medicine by an administrative decision called a 'compulsory (or government use) license’ to protect public health.

The patent application of sofosbuvir has been rejected by the EGPO, because it does not fulfill the patentability criteria of novelty and inventiveness, in accordance with the Egyptian law on intellectual property rights. The Key informant from EGPO explains the reason for patency rejection was due to the molecule sofosbuvir was known in advance and is not truly a novel molecule. In addition, the insufficiency of clinical trials conducted on patients of HCV infected with the genotype 4 that spread in Egypt and the need for a period of more than 6 months to ensure its safety and efficacy. It has been noted that no item of DAAs obtained patent protection, this gave the Egyptians the authority to produce generic versions of DAAs locally without IP-related restrictions.

### Local production of DAAs

The size of the pharmaceutical market in Egypt was about USD 2.3 billion in 2018 [50]. The local industry supplies 82% of the volume of the medicines consumed in Egypt, providing affordable medicines to the Egyptians. The remaining 18% are imported pharmaceuticals. Currently, the total number of licensed pharmaceutical manufacturers in Egypt is 152, in which 11 manufacture brand name medicines for multinational companies and ten manufacturers are producing Biopharmaceuticals in the country.

In 2007, the government issued a decision to adopt the WHO Good Manufacturing Practices for Pharmaceutical Products (GMP) as the Egyptian guide for good manufacturing standards. Pharmaceutical manufacturers of pharmaceutical products are requested by the Ministerial Decree no. 539/2007 to abide by this guideline [51]. Although the EDA is not internationally recognized as a “WHO-Listed Authority (WLA) [52]” and Egypt is not yet a member of the pharmaceutical inspection cooperation scheme (PIC/S), meaning certificates for Good Manufacturing Practices issued by EDA may not be recognized in other countries, the key informants confirmed that EDA has an appropriate organizational structure and an adequate number of qualified staff to guarantee that the manufacture, trade, and use of medicines are regulated effectively and to maintain that all activities and premises comply with GMP through inspecting manufacturers and assessing the safety, efficacy, and quality of medicines before marketing authorization. EDA conducts regular inspections on local manufacturing facilities, including site visits and random samples testing to ensure compliance with GMP. In case of non-compliance, EDA can instruct the marketing authorization holder to recall, destroy and/or re-export non-compliant pharmaceuticals. In case of significant violations, EDA can cancel the pharmaceutical marketing authorization or the pharmaceutical institution's license.

Since the IP-related restrictions of DAAs have been addressed in Egypt, the NRA granted local pharmaceutical companies’ permission to produce DAAs generics. As for January 2021, there are 44 local manufacturers in Egypt that are producing 67 generics of DAAs in Egypt.

It is important to note that collaboration between the government, civil society, and pharmaceutical companies led to improve patients’ access to affordable DAAs in Egypt. The treatment of DAAs was introduced into the national program during the third quarter of 2015, at more affordable prices, prompting a substantial scale-up of treatment. About two million individuals received HCV antiviral therapy up to 2018, mostly supported through government expenditure or health insurance. In 2018, Egypt launched one of the largest nationwide screening health campaigns “100 million healthy lives” under the directive of the Egyptian President. Egypt screened 35 million people for the hepatitis C virus (HCV) between October 2018 and March 2019 to supercharge the response to viral hepatitis. Mechanisms that have contributed to improving access to DAA in Egypt is shown in Table 3.

**Table 3:** Summary of the mechanisms that contributed to improve access to DAA in Egypt

Market authorization	Fast track registration of DAAs generics has been granted to the interested companies
	The DAAs have been exempted from the condition that they should be registered and distributed in the country of origin or the reference country for at least 1 year to accelerate the registration of DAAs
	Egypt enabled early market entry of DAAs generics
	The local pharmaceutical manufacturers have been allowed to register an unlimited number of DAAs (formerly, Egypt used the “Box” system in the authorization of Pharmaceuticals that limits the number of registered products to 12, in which the maximum number of registered generics for each brand product must not exceed 11 items)
Pricing	Multiple pricing policies were used to achieve low prices of DAAs that included external reference pricing in conjunction with price negotiation, and policies to promote the use of quality‑assured generics
	Regular price revisions are undertaken at a pre‑specified frequency
	DAAs medicines and their active ingredients were exempted from taxations
Intellectual property and patent protection	Law 82/2002 on intellectual property protection offers a wide range of flexibilities with the aim of protecting public access to medicines from drug monopolies and anti-competitive practices by patent holders
	The Egyptian Patent Office applies rigorous patent examination practices in the form of high patentability standards, only allowing medicines with ‘strong’ patent applications to be protected
	The DAAs didn’t get patent protection by the Egyptian Patent Office, this gave the local pharmaceutical manufacturers to produce generic versions of DAAs without IP-related restrictions
Local production	The government promotes and facilitates local production of HCV treatment
	Competition between producers of the generic versions of the DAAs reduced the price of these medicines, making it available and accessible to Egyptian patients at affordable prices
Other factors	Political will: The government established national guidelines for the treatment of chronic HCV, worked closely with several local and international companies to ensure adequate supply of DAAs, financially sponsored the treatment for all patients who are not covered by health insurance and provide free treatment to poor patients
	Community-based participation: Several civil societies contributed to HCV screening efforts, community-based education, and supporting the treatment costs for uninsured patients. The local media played a key role in raising awareness about HCV and promoting screening and treatment efforts

The study is subject to some limitations including the following:

First, the publications available on the regulation for HCV-DAAs were limited. To minimize this risk, information sources other than academic journals were systematically searched and used. The data source includes the interviews of key informants, national laws, presidential and ministerial decrees related to the regulation of pharmaceuticals. In addition to the web pages of the national regulatory authority and governmental websites.

Second, detailed information on the prices of DAAs was not easily available from the public domain of the countries in the middle east and north Africa to compare it with the prices in Egypt. Therefore, we have selected the following neighboring Arab countries: Jordan, Lebanon, Morocco, Saudi Arabia, and the United Arab Emirates, because their prices are publicly available on the government’s websites. A simple comparison of product price by country is not a good means for comparing the efficiency of pharmaceutical policy and regulation, and for proposing concrete policy recommendations. However, this is expected minimal impact on the aim of the study, a more comprehensive understanding of the global impacts of access to HCV-DAAs may require further research on the impacts of regulatory harmonization on the pricing of these items.

## Conclusions

History records very rare opportunities to eliminate a chronic infection. With the availability of new, effective DAAs to treat Hepatitis C and the political will to eliminate HCV, Egypt undertook an ambitious elimination model program to fulfill the WHO’s elimination goals and offered treatment on a scale that can reduce the prevalence to a level that equals the elimination of the disease.

The Egyptian regulatory system contributes to making safe and effective DAAs available at affordable prices through a fast-track marketing authorization process of new effective DAAs, price negotiation to guarantee lower prices of brand DAAs, using TRIP’s flexibility to prohibit or revoke patency to promote local production of patented drugs at lower prices. Egypt succeeded to introduce low-cost generic drugs with the same efficacy and safety of branded medicines into the market and significant progress has been made in creating a manufacturing base to safeguard supply at prices currently below the internationally estimated “best price” benchmark. The study findings suggest that the current Egyptian pharmaceutical approaches and regulations have a tremendous part in improving the availability and affordability of medicines towards the elimination of hepatitis C in Egypt.

## Supplementary Information

**Additional file 1: Diagram S1.** Methodology used in this study. **Table S1.** List of National Laws related to regulation of pharmaceuticals in Egypt. **Table S2.** List of Presidential Decrees related to regulation of pharmaceuticals in Egypt. **Table S3.** List of Ministerial Decrees related to regulation of pharmaceuticals in Egypt. **Table S4.** List of web pages of Key Stakeholder Organizations (Egypt). **Table S5.** Web pages of Organizations (Regional and International Level). **Table S6.** Summary of the type of interviewees by profession/Institution. **Table S7.** Interview guide. **Table S8.** List of registered DAAs in Egypt.

## Data Availability

All data for analysis in this review is in the public domain.

## References

[1] Bhamidimarri KR, Satapathy SK, Martin P (2017). Hepatitis C virus and liver transplantation. Gastroenterol Hepatol (N Y).

[2] World Health Organization (WHO). Hepatitis C fact sheet. Updated 27 July 2020. https://www.who.int/news-room/fact-sheets/detail/hepatitis-c. Accessed 9 Apr 2021.

[3] Kandeel A, Genedy M, El-Refai S, Funk AL, Fontanet A, Talaat M (2017). The prevalence of hepatitis C virus infection in Egypt 2015: implications for future policy on prevention and treatment. Liver Int.

[4] Kouyoumjian SP, Chemaitelly H, Abu-Raddad LJ (2018). Characterizing hepatitis C virus epidemiology in Egypt: systematic reviews, meta-analyses, and meta-regressions. Sci Rep.

[5] Egypt’s Viral Hepatitis Program. Burden and response: an economic analysis 2017. The World Bank’s. http://documents.worldbank.org/curated/en/972381517328583384/pdf/123066-WP-PUBLIC-P157533-Burden-and-response-An-Economic-Analysis.pdf. Accessed 9 Apr 2021.

[6] Tsai SM, Kao JT, Tsai YF (2016). How hepatitis C patients manage the treatment process of pegylated interferon and ribavirin therapy: a qualitative study. BMC Health Serv Res.

[7] Sandmann L, Schulte B, Manns MP, Maasoumy B (2019). Treatment of chronic hepatitis C: efficacy, side effects and complications. Visc Med.

[8] Holmes JA, Rutledge SM, Chung RT (2019). Direct-acting antiviral treatment for hepatitis C. Lancet.

[9] Bourlière M, Bronowicki JP, de Ledinghen V, Hézode C, Zoulim F, Mathurin P (2015). Ledipasvir-sofosbuvir with or without ribavirin to treat patients with HCV genotype 1 infection and cirrhosis non-responsive to previous protease-inhibitor therapy: a randomised, double-blind, phase 2 trial (SIRIUS). Lancet Infect Dis.

[10] Arab Republic of Egypt, Official Gazette. Law No. 151 of 2019 for establishing the Egyptian Drug Authority. https://manshurat.org/node/61255. Accessed 9 Apr 2021.

[11] Egyptian Drug Authority. Pharmacy Law 127 for 1955. [Online]. http://www.eda.mohp.gov.eg/Files/95_Pharmacy_law.pdf. Accessed 9 Apr 2021.

[12] State of information service. https://www.sis.gov.eg/section/0/4496?lang=en-us. Accessed 9 Apr 2021.

[13] Waheed Y, Siddiq M, Jamil Z, Najmi MH (2018). Hepatitis elimination by 2030: progress and challenges. World J Gastroenterol.

[14] Global Health Sector Strategy on Viral Hepatitis, 2016–2021. Geneva: World Health Organization; 2015. https://www.who.int/hepatitis/strategy2016-2021/ghss-hep/en/. Accessed 9 Apr 2021.

[15] Amin MEK, Nørgaard LS, Cavaco AM, Witry MJ, Hillman L, Cernasev A (2020). Establishing trustworthiness and authenticity in qualitative pharmacy research. Res Soc Adm Pharm.

[16] Palinkas LA, Horwitz SM, Green CA, Wisdom JP, Duan N, Hoagwood K (2015). Purposeful sampling for qualitative data collection and analysis in mixed method implementation research. Adm Policy Ment Health.

[17] Cresswell JW, Plano Clark VL (2011). Designing and conducting mixed method research.

[18] Wanis H, Zaheer-Ud-Din B (2015). Chapter 4—pharmaceutical pricing in Egypt. Pharmaceutical prices in the 21st century.

[19] Egyptian Pricing Decree no499/2012. Egyptian Drug Authority. In Arabic at http://www.eda.mohp.gov.eg/Files/474_499.pdf. Accessed 9 Apr 2021.

[20] Lashen SA, Shamseya MM, Madkour MA, Aboufarrag GA (2019). Tolerability and effectiveness of generic direct-acting antiviral drugs in eradication of hepatitis C genotype 4 among Egyptian patients. Liver Int.

[21] Abozeid M, Alsebaey A, Abdelsameea E, Othman W, Elhelbawy M, Rgab A (2018). High efficacy of generic and brand direct acting antivirals in treatment of chronic hepatitis C. Int J Infect Dis.

[22] Ndomondo-Sigonda M, Miot J, Naidoo S, Dodoo A, Kaale E (2017). Medicines regulation in Africa: current state and opportunities. Pharm Med.

[23] US Food and Drug Administration (FDA). Learn about FDA, its mission, history, how it's organized, and more. https://www.fda.gov/. Accessed 9 Apr 2021.

[24] European Medicines Agency. The European Medicines Agency’s contribution to science, medicines and health in 2019. https://www.ema.europa.eu/en/documents/annual-report/2019-annual-report-european-medicines-agency_en.pdf. Accessed 9 Apr 2021.

[25] Egyptian Drug Authority. Ministry Decree 425/2015. [Online]. http://www.eda.mohp.gov.eg/Files/766_425_2015.pdf. Accessed 9 Apr 2021.

[26] Li G, De Clercq E (2017). Current therapy for chronic hepatitis C: the role of direct-acting antivirals. Antivir Res.

[27] Burstow NJ, Mohamed Z, Gomaa AI, Sonderup MW, Cook NA, Waked I (2017). Hepatitis C treatment: where are we now?. Int J Gen Med.

[28] Gritsenko D, Hughes G (2015). Ledipasvir/sofosbuvir (Harvoni): improving options for hepatitis C virus infection. Pharm Ther.

[29] Keating GM, Vaidya A (2014). Sofosbuvir: first global approval. Drugs.

[30] Montgomery M, Ho N, Chung E, Marzella N (2016). Daclatasvir (Daklinza): a treatment option for chronic hepatitis C infection. P T.

[31] Raedler LA. Once-a-day Harvoni (ledipasvir plus sofosbuvir), a new oral combination for the treatment of patients with genotype 1 chronic hepatitis C infection. Am Health Drug Benefits. 2015;8(Spec Feature):54–8. PMC4665066 26629267

[32] Afdhal N, Reddy KR, Nelson DR, Lawitz E, Gordon SC, Schiff E (2014). Ledipasvir and sofosbuvir for previously treated HCV genotype 1 infection. N Engl J Med.

[33] Zhang S, Bastian ND, Griffin PM (2015). Cost-effectiveness of sofosbuvir-based treatments for chronic hepatitis C in the US. BMC Gastroenterol.

[34] Egyptian Initiative for Personal Rights. HCV treatment in Egypt: why cost remains a challenge? November 2014. https://www.eipr.org/sites/default/files/pressreleases/pdf/hcv_treatment_in_egypt.pdf. Accessed 9 Apr 2021.

[35] Hill A, Simmons B, Gotham D, Fortunak J (2016). Rapid reductions in prices for generic sofosbuvir and daclatasvir to treat hepatitis C. J Virus Erad.

[36] Iyengar S, Tay-Teo K, Vogler S, Beyer P, Wiktor S, de Joncheere K (2016). Prices, costs, and affordability of new medicines for hepatitis C in 30 countries: an economic analysis. PLoS Med.

[37] World Bank. World Bank list of economies (June 2020). https://datahelpdesk.worldbank.org/knowledgebase/articles/906519. Accessed 9 Apr 2021.

[38] Arab Republic of Egypt, Egyptian Drug Authority. http://196.46.22.218/EDASearch/SearchRegDrugs.aspx. Accessed 9 Apr 2021.

[39] Hashemite Kingdom of Jordan. Jordan Food and Drug Administration. http://jfda.jo/Pages/viewpage.aspx?pageID=184. Accessed 9 Apr 2021.

[40] Republic of Lebanon, Ministry of Public Health. https://moph.gov.lb/en/Pages/3/3101/drugs-public-price-list-. Accessed 9 Apr 2021.

[41] Kingdom of Morocco, Ministry of Health. https://www.sante.gov.ma/sites/Ar/medicaments/Pages/PMed.aspx. Accessed 9 Apr 2021.

[42] Kingdom of Saudi Arabia, Saudi Food and Drug Authority. https://www.sfda.gov.sa/en/drug/search/Pages/default.aspx. Accessed 9 Apr 2021.

[43] United Arab Emirates, Ministry of Health and Prevention. https://www.mohap.gov.ae/en/services/request-for-a-price-list-of-registered-medications. Accessed 9 Apr 2021.

[44] Abdellatif L, Zaky M (2015). Characteristics of private markets and accessibility of small and medium enterprises to public procurement markets: pharmaceuticals in Egypt. J Public Procure.

[45] Gericke CA, Britain K, Elmahdawy M, Elsisi G, van Ginneken E, Busse R (2018). Health system in Egypt. Health care systems and policies. Health services research.

[46] Arab Republic of Egypt. 2002. Law 82/2002 on the protection of intellectual property rights. Official Gazette (22), 2 June 2002. http://www.wto.org/english/thewto_e/minist_e/min01_e/mindecl_trips_e.htm. Accessed 9 Apr 2021.

[47] World Intellectual Property Organization. Agreement between the Egyptian Academy of Scientific Research and Technology and the International Bureau of the World Intellectual Property Organization in relation to the functioning of the Egyptian Patent Office as an International Searching Authority and International Preliminary Examining Authority under the Patent Cooperation Treaty. https://www.wipo.int/export/sites/www/pct/en/texts/agreements/ag_eg.pdf. Accessed 9 Apr 2021.

[48] Boldrin M, Levine DK (2013). The case against patents. J Econ Perspect.

[49] 't Hoen E, Passarelli CA. The role of intellectual property rights in treatment access: challenges and solutions. Curr Opin HIV AIDS. 2013;8(1):70–4. 10.1097/COH.0b013e32835b6e5a 23201855

[50] Egypt Pharmaceuticals & Healthcare Report, 2019. https://nations-emergentes.org/wp-content/uploads/2019/02/egypt-pharmaceutical-survey.pdf. Accessed 9 Apr 2021.

[51] Egyptian Drug Authority. Ministry Decree 539/2007. [Online]. http://www.eda.mohp.gov.eg/Files/105_Minister_Decree_539_Both.pdf. Accessed 9 Apr 2021.

[52] Broojerdi AK, Sillo HB, Dehaghi RO, Ward M, Refaat M, Parry J (2020). The World Health Organization global benchmarking tool an instrument to strengthen medical products regulation and promote universal health coverage. Front Med.

