# Supplementary material for: Pharmaceutical policies and regulations of oral antiviral drugs for treatment of hepatitis C in Egypt—case study
Source: J Pharm Policy Pract. 2021 Dec 16;14:106. doi: 10.1186/s40545-021-00389-6 (PMC8674831; doi:10.1186/s40545-021-00389-6)
Supplement: Supplementary file 1 — Additional file 1: Diagram S1. Methodology used in this study. Table S1. List of National Laws related to regulation of pharmaceuticals in Egypt. Table S2. List of Presidential Decrees related to regulation of pharmaceuticals in Egypt. Table S3. List of Ministerial Decrees related to regulation of pharmaceuticals in Egypt. Table S4. List of web pages of Key Stakeholder Organizations (Egypt). Table S5. Web pages of Organizations (Regional and International Level). Table S6. Summary of the type of interviewees by profession/Institution. Table S7. Interview guide. Table S8. List of registered DAAs in Egypt. [file 40545_2021_389_MOESM1_ESM.docx]

Diagram S1 Methodology used in this study

Table S1: List of National Laws related to regulation of pharmaceuticals in Egypt

| Law No. 151/2019 | Establishing the Egyptian Drug Authority |
| --- | --- |
| Law No. 2/2018 | Establishing the comprehensive health insurance programme |
| Law No. 206/2017 | Regulating the advertising of health products and services |
| Law No. 82/2002 | Protection of Intellectual Property Rights. |
| Law No. 47/1969 | Establishment of pharmacists’ Syndicate |
| Law No. 13/1964 | Preparation of medicines and pharmaceuticals in pharmacies, under trade names or for trading purpose |
| Law No. 113/1962 | Regulating the importation, manufacturing and trade of medicines, medical requisites and chemicals. |
| Law No. 212/1960 | Regulating the trade of medicines, chemicals and medical requisites |
| Law No. 182/1960 | Drug control and organization of narcotic use |
| Law No. 9/1959 | Importing and exporting |
| Law No. 21/1958 | Organization of industry |
| Law No. 127/1955 (amended by Law 253/1955, Law 7/1956, Law 360/1956, Law 61/1959, Law 44/1982, Law 14/1984, Law 81/1997, Law 167/1998) | Regulation of pharmacies (Pharmacy Law). |
| Law No. 163/1950 | Compulsory pharmaceutical pricing and profits |
| Law No. 48/1941 (amended by Law 106/1980, Law 281/1994) | Prevention of fraud and deception |

Table S2: List of Presidential Decrees related to regulation of pharmaceuticals in Egypt

| Decree No. 18/2020 | Establishment of the Egyptian Drug Authority |
| --- | --- |
| Decree No. 242/1996 | Organization of the Ministry of Health |
| Decree No. 398/1995 | Establishment of the National Organization for Research and Control of Biologics (NORCB) |
| Decree No. 382 of 1976 | Establishment of the National Authority for Drug Control and Research (NODCAR) |
| Decree No. 94/1972 | Establishment of the Egyptian General Organization for Biological Products and Vaccines |
| Decree No. 113/1962 | Regulating the importation, manufacturing and trade of medicines, medical requisites and chemicals. |

Table S3: List of Ministerial Decrees related to regulation of pharmaceuticals in Egypt

| Decree No. 116/2017 | Importation of pharmaceuticals from reference and non-reference countries |
| --- | --- |
| Decree No. 29/2016 | Pharmaceutical Track and Trace |
| Decree No. 734/2016 | Regulations of Good Clinical Practice (GCP Guidelines) |
| Decree No. 425/2015 | Registration of pharmaceuticals for human beings |
| Decree No. 499/2012 | Pricing of pharmaceuticals |
| Decree No. 575/2012 | Registration of pharmaceutical products |
| Decree No. 622/2012 | Exportation of pharmaceuticals |
| Decree No. 640/2012 | Fees of services provided by CAPA |

| Decree No. 172/2011 | Regulation of psychotropic medicines |
| --- | --- |
| Decree No. 2/2010 | Pharmacovigilance guidelines |
| Decree No. 373/2009 | Pricing of pharmaceuticals. |
| Decree No. 25/2009 | Regulations of warehousing and distribution of pharmaceuticals businesses |
| Decree No. 296/2009 | Registration of human drugs |
| Decree No. 297/2009 | Registration of biologicals |
| Decree No. 380/2009 | Specifications of pharmacies and stores |
| Decree No. 539/2007 | Egyptian code for good manufacturing practices for pharmaceuticals |
| Decree No. 540/2007 | Withdrawal and prohibition of pharmaceutical products do not comply with specifications |
| Decree No. 435/2006 | Good manufacturing practices for pharmaceuticals for biologicals |
| Decree No. 76/2000 | Regulating advertising of medicines and pharmaceutical products and dietary supplements |
| Decree No. 38/1999 | Mandatory continuity of production by pharmaceutical manufacturing facilities |
| Decree No. 91/1999 | Restriction of advertising for pharmaceuticals without prior approval. |
| Decree No. 487/1985 | Regulations for controlled drugs and substances |
| Decree No. 429-1976 | Regulating scientific offices |
| Decree No. 645/2018 | Registration of pharmaceutical products |

**Table S4: List of** **web pages of Key Stakeholder Organizations (Egypt)**

| Type of Organization | Identified organizations | address |
| --- | --- | --- |
| Regulatory authority | Egyptian Drug Authority (including Central Administration of Pharmaceutical Affairs) | <http://www.eda.mohp.gov.eg/> |
|  | National Organization for Drug & Control Research | <http://www.nodcar.eg.net/main/> |
|  | National Organization for Research and Control of Biologicals | <http://norcb.gov.eg/> |
| Public | Egyptian Ministry of Health and Population | <http://www.mohp.gov.eg/> |
|  | Egyptian Patent Office | <http://www.egypo.gov.eg/> |
|  | State of Information Service | <https://www.sis.gov.eg/?lang=en-us> |
|  | The Academy of Scientific Research & Technology (ASRT) | <http://www.asrt.sci.eg/index.php> |
|  | Ministry of Trade & Industry | <http://www.mti.gov.eg/english/Pages/default.aspx> |
|  | Ministry of Finance | <http://www.mof.gov.eg/english/pages/home.aspx> |
|  | Amiri Press/ Official Gazette | <http://www.alamiria.com/> |
| Private non-profit | Egyptian Pharmacist Syndicate | <https://eps-eg.org/> |
|  | The Egyptian society of hepatology, gastroenterology and infectious diseases | <http://www.eshgid.com/index.php> |
| Private industrial sector | Pharmaceutical Egyptian Association | <http://www.fei.org.eg/index.php/en/> |
|  | Pharmed Health Care | <https://pharmedhc.com/> |
|  | Zeta Pharma | <https://www.zetapharma.net/> |

Table S5: Web pages of Organizations (Regional and International Level)

| **Institutional stakeholders** | **Identified organizations** | **Website** |
| --- | --- | --- |
| Regulatory Authority | The European Medicines Agency (EMA) | <https://www.ema.europa.eu/en> |
|  | The United State Food and Drug Administration (FDA) | <https://www.fda.gov/> |
|  | Jordan Food and Drug Administration (JFDA) | <http://www.jfda.jo/> |
|  | Saudi Food and Drug Authority | <https://www.sfda.gov.sa/> |
| Public | Lebanon Ministry of Public Health | <https://moph.gov.lb/> |
|  | Morocco Ministry of Health | <https://www.sante.gov.ma/> |
|  | UAE Ministry of Health and Prevention | <https://www.mohap.gov.ae/> |
| International organization  (Public) | World Health Organization | <https://www.who.int/> |
|  | World Trade Organization | <https://www.wto.org/> |
|  | World Intellectual Property Organization | <https://www.wipo.int/> |
|  | United Nations Industrial Development Organization | <https://www.unido.org/> |
|  | World Bank | <https://www.worldbank.org/> |
|  | Unitaid | <https://unitaid.org/#en> |
| International organization  (Private: non-profit) | International Council for Harmonization of Technical Requirements for Pharmaceuticals (ICH) | <https://www.ich.org/> |
|  | Pharmaceutical Inspection Convention and Pharmaceutical Inspection Co-operation Scheme | <https://picscheme.org/> |
|  | International Pharmaceutical product Policy Consortium | <https://idpc.net/> |
|  | World Hepatitis Alliance | <https://www.worldhepatitisalliance.org/> |
|  | Hepatitis B and C Public Policy Association | <http://www.hepbcppa.org/> |
|  | Hepatitis C trust | <http://www.hepctrust.org.uk/> |
| Pharmaceutical companies | International Federation of Pharmaceutical Manufacturers & Associations | <https://www.ifpma.org/> |
|  | Gilead Sciences, Inc | <https://www.gilead.com/> |
|  | Bristol-Myers Squibb Company | <https://www.bms.com/> |
|  | AbbVie | <https://www.abbvie.com/> |
|  | Merck & Co. | <https://www.merck.com/index.html> |
|  | Janssen | <https://www.janssen.com/> |

Table S6: Summary of the type of interviewees by profession/ Institution

| **Institution** | **Number of**  **interviewees** |
| --- | --- |
| Central Administration of Pharmaceutical Affair (CAPA)- Registration | 2 |
| Central Administration of Pharmaceutical Affair (CAPA)- Pricing | 2 |
| Member of pricing committee | 1 |
| Central Administration of Pharmaceutical Affair (CAPA)- inspection | 1 |
| Egyptian pharmacovigilance center | 1 |
| Egyptian Drug information center | 1 |
| Independent –Senior ex-CAPA | 1 |
| Ministry of Health and Population - Procurement of Medicines Department | 1 |
| Egyptian Patent Office/Academy for Scientific Research | 2 |
| Pharmaceutical Industry Chamber | 3 |
| Local pharmaceutical manufacturers | 5 |
| Key Opinion Leader Clinicians using DAAs in treating patients | 2 |
| Pharmacy syndicate | 4 |
| Academia and pharmaceutical research | 3 |
| Civil Society (patient’s rights) | 2 |
| Total | 31 |

Table S7: Interview guide

| **1. Introduction (10 minutes)** |
| --- |
| 1.1 Self-introduction  - I want to thank you for taking the time to meet with me today.  - My name is … |
| **1.2 Purpose of the study**  - For my research, I would like to talk to you about your experience with the pharmaceutical regulation of medicine in Egypt, especially on Direct-Acting Antivirals (DAAs) for treatment of Hepatitis C virus (HCV) infection.  - HCV infection is the most challenging public health problem in Egypt where the prevalence is the highest in the world. The revolution in hepatitis C treatment with DAAs has, for the first time, provided an opportunity for widespread scale-up of curative treatment. However, to eliminate HCV, the bottom line of the challenge ahead is how to provide access to such high-cost medicines that used to treat HCV infection.  - This study aims to answer the question of “whether the current government policies for oral antiviral drugs regulation, specially DAAs are adequate to eliminate HCV in Egypt”. To answer this question, we examine four independent variables:  1-The current regulatory requirements and procedures by the Egyptian government authorities involved in granting market authorization of DAAs.  2- The present pricing policy and mechanisms for pricing medicines and DAAs.  3- The national intellectual property and patent protection regulation in Egypt and its relation to access to DAAs products.  4. Local production of low-cost generic DAAs and the quality of generic DAAs in Egypt.  - Therefore, this study will describe the current pharmaceutical regulation of the new oral antiviral drugs to treat hepatitis C in Egypt and will examine its implication and contribution to eliminate HCV. As there are limited studies on the role of efficient regulatory mechanisms in facilitating greater access to HCV treatment, this study will provide the evidence to support the importance of effective regulations in improving access to oral viral drug and elimination of HCV.  - Consequently, your input is important since you have been (directly/indirectly) involved in the  process, and has substantial knowledge on this topic. |
| 1.3 Informed consent  - The interview should take less than an hour. I will be taking some notes during the session,  but I will also be recording the session because I do not want to miss any of your comments, if that is fine with you.  - All responses will be kept confidential. This means that your interview response would not be  shared with anyone except myself that any information included in the study would be  anonymized. Also, you do not have to talk about anything that you do not want to, and you  may end the interview at any time.  - Are there any questions about what I have just explained?  - Are you willing to participate in this interview?  - If so, please do sign the informed consent form |
| **2. About yourself and your organization (5 minutes)**  1. Could you describe your role in your organization? |
| 3. Technical questioned based on the area of expertise (30-40 minutes) |
| **I. Marketing Authorization (Registration)of DAAs**  **1. Please describe the regulatory authority in Egypt?**  a. Organogram and functions of the National Medicines Regulatory Authority  b. Duty of each department  c. Laws and legislations  **2.** Please illustrate the steps of registration medicines in Egypt?  a. Procedures for assessors on how to assess applications submitted for registration  a. Laws, decrees and guidelines related to registration of medicines in Egypt  b. Time limit for the assessment of a Marketing Authorization application (months)  c. Renewal procedure  d. Appeal mechanism  3. Please describe technical committees involved in marketing authorization process?  a. Criteria for selecting the members (scientific and technical expertise)  b. TORs  b. Decision-making process  c. Declaration of COI  4. Is registration of DAAs differ from registration of other medicines. Please explain?  **5. Do you think the process of registration of DAAs ensures quality, safety and efficacy? Why?**  **6. Please describe the key obstacles experienced during registration of DAAs?**  **7. What lessons should be drawn from the experiences of registration DAAs?**  **8. What policy would you recommend for improving marketing authorization and access to DAAs?** |
| II. Pricing and reimbursement of DAAs  1. Please elucidate the procedure of pricing medicines in Egypt?  a. Type of pricing implemented: compulsory pricing; external reference pricing; internal reference pricing; value-based pricing; negotiation, cost-plus pricing …etc.  b. Laws, decrees and guidelines related to pricing of medicines in Egypt  c. How the decision made are being communicated and implemented  2. Please describe the pricing committees?  a. Scientific and technical expertise and their TORs  b. Pricing committee has the scientific capacity to assess pharmacoeconomic studies to establish a price to the value of product  C. Decision-making process  D. Declaration of COI  3. Is the process of pricing transparent and ensure affordability of medicines?  4. Can the pharmaceutical company appeal if they felt the price wasn’t fair, if such who are the appeal committee?  5. Is pricing of DAAs differ from pricing of other medicines. Please explain?  6. Please describe the key obstacles experienced during pricing of DAAs?  7. What lessons should be drawn from the experiences of pricing DAAs in Egypt?  8. What policy would you recommend for improving pricing and access to DAAs? |
| III. Medicines trade and intellectual property  1. Please elaborate on the national laws and guidelines related to the protection of intellectual property rights in Egypt and their implementations?  a. National legislation has been modified to implement the TRIPS Agreement  b. Current laws contain (TRIPS) flexibilities and safeguards  c. Are there legal provisions for data exclusivity for medical products  d. Legal provisions exist for patent extension  2. Please describe the activities performed by the Egyptian Patent Office  a. Developing property legislations  b. Registering patent application for local & foreign inventions  c. Granting and issuing patents to protect the rights of Egyptian & foreigner inventors  d. Transferring the technological information from the patents and provide it to the specialists in order to develop their works as well as developing local industries  3. Please describe the Egyptian experience related to the patency of DAAs?  4. What lessons should be drawn from the experiences related to the patency of DAAs?  5. What policy would you recommend for preserving using the TRIPS flexibilities and encourage investment in the local production of DAAs? |
| IV. Local Manufacturing of DAAs  1. Please list the total number of licensed pharmaceutical manufacturers in your country in terms of ownership and how many of them are producing DAAs  a. Governmental manufacturers  b. Private (national) manufacturers  c. Private (multinational) manufacturers:  2. what are the DAAs that are locally manufactured?  3. Is there any local manufacturer produce active ingredients of DAA?  4. Does the Government have a vision and strategic plan for the promoting local production of medical products, especially in the field of R&D promotion for DAAs DAAs. Please clarify?  5. How the government ensure high quality of locally produced DAAs?  6. What are the challenges facing local manufacturers to produce DAAs?  7. What are your recommendations to ensure high quality of locally produced DAAs?  8. How do you perceive the market for DAAs in Egypt in the next 5 years? Market Growth and profitability? |
| 4. Ending the interview (5 minutes)  Is there anything more you would like to add?  - I will be the one who will be analyzing the information you have given to me. I am hoping to  submit a draft paper by June 2020. I will be happy to send you a copy to review at that  time if you are interested.  - Thank you for your time. |

Table S8: List of registered DAAs in Egypt

| Generic Name | Trade Name | Strength & dosage form | Marketing Company | Price 1 Box LE | Package Size | Registration Date |  |
| --- | --- | --- | --- | --- | --- | --- | --- |
| **Protease inhibitors (NS5A inhibitors)** | | | | | | | |
|  |  |  |  |  |  |  |  |
| 1. Daclatasvir | Daklinza®  Clatazev® | 60 mg tablet | Bristol-Myers Squibb | EGP 8000 | 28 | 8/2015 |  |
| 2. Daclatasvir | daclavirocyrl® | 60 mg tablet | Marcyrl pharmaceuticals | EGP 120 | 28 | 10/2015 |  |
| 3. Daclatasvir | Daklanork® | 60 mg tablet | Mash Premiere | EGP 120 | 28 | 11/2015 |  |
| 4. Daclatasvir | Augidacla® | 60 mg tablet | AUG Pharma | EGP 120 | 28 | 2/2016 |  |
| 5. Daclatasvir | Daktavira® | 60 mg tablet | European egyptian pharmaceutical industries | EGP 120 | 28 | 2/2016 |  |
| 6. Daclatasvir | daclavirdin® | 60 mg tabet | Eva Pharma | EGP 120 | 28 | 3/2016 |  |
| 7. Daclatasvir | Javidacla® | 60 mg tablet | Multicare Egypt for Pharmaceutical Industries | EGP 200 | 28 | 3/2016 |  |
| 8. Daclatasvir | Daclahepex® | 66 mg tablet | Global Pharmaceutical Industries | EGP 120 | 28 | 9/2016 |  |
| 9. Daclatasvir | Zetaciver® | 60 mg tablet | Zeta Pharma | EGP 120 | 28 | 9/2016 |  |
| 10. Daclatasvir | Daclatasvir-Uccma® | 30 mg tablet | United Company | EGP 73 | 28 | 2/2017 |  |
| 11. Daclatasvir | Daclatasvir-Uccma® | 60 mg tablet | United Company | EGP 120 | 28 | 2/2017 |  |
| 12. Daclatasvir | Daclavir® | 60 mg tablet | Dawood pharma trade | EGP 120 | 28 | 2/2017 |  |
| 13. Daclatasvir | Andodaclata® | 60 mg tablet | Al Andalous for pharmaceutical industries | EGP 120 | 28 | 8/2018 |  |
| 14. Daclatasvir | Dacladazin® | 60 mg tablet | EIPICO | EGP 120 | 28 | 3/2019 |  |
| 15. Daclatasvir | Dacladova® | 60 mg tablet | Hikma Pharmaceuticals industries | EGP 120 | 28 | 3/2019 |  |
|  |  |  |  |  |  |  |  |
| **Nucleotide/nucleoside and nonnucleoside polymerase inhibitors (NS5B inhibitors)** | | | | | | | |
|  |  |  |  |  |  |  |  |
| 1. Sofosbuvir | Sovaldi ® | 400 mg tablet | " Gilead access program", IBIS Pharma | EGP 4,840 | 28 | 7/2014 |  |
| 2. Sofosbuvir | Sofolanork | 400 mg tablet | Mash Premiere | EGP 900 | 28 | 10/2014 |  |
| 3. Sofosbuvir | Mpiviropack | 400 mg tablet | Marcyrl Pharmaceuticals | EGP 900 | 28 | 12/2014 |  |
| 4. Sofosbuvir | Serinospirevir | 400 mg tablet | SAJA Pharmaceuticals Egypt | EGP 900 | 28 | 12/2014 |  |
| 5. Sofosbuvir | Sofosbuvir I.P.M.C | 400 mg tablet | I.P.M.C |  | 28 | 1/2015 |  |
| 6. Sofosbuvir | Tigaglor | 400 mg tablet | Aseya Mary | EGP 900 | 28 | 1/2015 |  |
| 7. Sofosbuvir | Heterosofir | 400 mg tablet | Pharmed health care | EGP 900 | 28 | 2/2015 |  |
| 8. Sofosbuvir | Sofosbuvir-Pharco B International | 400 mg tablet | Pharco B International |  | 28 | 2/2015 |  |
| 9. Sofosbuvir | Sofosbuvir-Biomed | 400 mg tablet | BIOMED |  | 28 | 2/2015 |  |
| 10. Sofosbuvir | Hopforhep | 400 mg tablet | Global NAPI Pharmaceuticals - GNP | EGP 2,403 | 28 | 3/2015 |  |
| 11. Sofosbuvir | Sofocivir | 400 mg tablet | Zeta Pharma | EGP 900 | 28 | 3/2015 |  |
| 12. Sofosbuvir | Sofosbuvir-IDI | 400 mg tablet | IDI |  | 28 | 3/2015 |  |
| 13. Sofosbuvir | Augispov | 400 mg tablet | AUG Pharma | EGP 900 | 28 | 4/2015 |  |
| 14. Sofosbuvir | Sofovirotal | 400 mg tablet | FUTURE PHARMACEUTICAL | EGP 900 | 28 | 4/2015 |  |
| 15. Sofosbuvir | Nucleobuvir | 400 mg tablet | Eva Pharma | EGP 900 | 28 | 7/2015 |  |
| 16. Sofosbuvir | Sofohep | 400 mg tablet | Sabaa International Company | EGP 900 | 28 | 7/2015 |  |
| 17. Sofosbuvir | Sofozav | 400 mg tablet | Hikma Pharmaceuticals | EGP 900 | 28 | 10/2015 |  |
| 18. Sofosbuvir | Andohepasuvir | 400 mg tablet | al andalous for pharmaceutical industries | EGP 900 | 28 | 12/2015 |  |
| 19. Sofosbuvir | Corcihepafect | 400 mg tablet | Star kamed for Medical Supplies | EGP 900 | 28 | 12/2015 |  |
| 20. Sofosbuvir | Episovir | 400 mg tablet | EIPICO | EGP 900 | 28 | 12/2015 |  |
| 21. Sofosbuvir | Sobosuvimec | 400 mg tablet | MEMPHIS | EGP 900 | 28 | 12/2015 |  |
| 22. Sofosbuvir | Sofomerase | 400 mg tablet | Amoun | EGP 900 | 28 | 12/2015 |  |
| 23. Sofosbuvir | Sofoplatin | 400 mg tablet | Dawood Pharma | EGP 900 | 28 | 12/2015 |  |
| 24. Sofosbuvir | Sofolorocy | 400 mg tablet | Actogenix | EGP 900 | 28 | 12/2015 |  |
| 25. Sofosbuvir | Sofosbuvir - Pharmaserve Medical | 400 mg tablet | Pharma Serve Medical |  | 28 | 12/2015 |  |
| 26. Sofosbuvir | Virunator | 400 mg tablet | Tabuk medical | EGP 900 | 28 | 12/2015 |  |
| 27. Sofosbuvir | Magicbuvir | 400 mg tablet | Magic Pharma | EGP 900 | 28 | 1/2016 |  |
| 28. Sofosbuvir | Soforoyal | 400 mg tablet | Royal Link Pharma | EGP 900 | 28 | 2/2016 |  |
| 29. Sofosbuvir | Sofodelevier | 400 mg tablet | D.B.K | EGP 900 | 28 | 2/2016 |  |
| 30. Sofosbuvir | Sofosbuvir-UCCMA | 400 mg tablet | UCCMA | EGP 900 | 28 | 2/2016 |  |
| 31. Sofosbuvir | Myhep | 400 mg tablet | One Pharma Tech | EGP 450 | 28 | 3/2016 |  |
| 32. Sofosbuvir | Kemisofo | 400 mg tablet | Chemipharm Pharmaceutical Industries |  | 28 | 4/2016 |  |
| 33. Sofosbuvir | Naplexovir | 400 mg tablet | Naplex Pharmaceutical | EGP 900 | 28 | 6/2016 |  |
| 34. Sofosbuvir | Sofosbuvir-MUP | 400 mg tablet | MUP | EGP 900 | 28 | 6/2016 |  |
| 35. Sofosbuvir | Sofoticlude | 400 mg tablet | HR INC | EGP 900 | 28 | 9/2016 |  |
| 36. Sofosbuvir | Pentasovir | 400 mg tablet | Penta Pharma Egypt | EGP 900 | 28 | 12/2016 |  |
| 37. Sofosbuvir | Sofosbuvir - Atco | 400 mg tablet | ATCO Pharma |  | 28 | 3/2017 |  |
| 38. Sofosbuvir | Grateziano | 400 mg tablet | European egyptian pharmaceutical industries | EGP 900 | 28 | 12/2019 |  |
|  |  |  |  |  |  |  |  |
| **Combinations** | | | | | | | |
|  | | | | | | | |
| 1. Sofosbuvir, Ledipasvir | Harvoni® | (400, 90) mg tablet | Gilead " Gilead access program", EVA Pharma | EGP 5,500 | 28 | 10/2015 |  |
| 2. Sofosbuvir, Ledipasvir | Sofolanork plus | (400, 90) mg tablet | Mash Premiere |  | 28 | 10/2015 |  |
| 3. Sofosbuvir, Ledipasvir | Sofolorocy Plus | (400, 90) mg tablet | Actogenix | EGP 1,100 | 28 | 10/2015 |  |
| 4. Sofosbuvir, Ledipasvir | Heterosofir Plus | (400, 90) mg tablet | Pharmed health care | EGP 1,500 | 28 | 11/2015 |  |
| 5. Sofosbuvir, Ledipasvir | mpiviropack plus | (400, 90) mg tablet | Marcyrl pharmaceuticals | EGP 1,500 | 28 | 1/2016 |  |
| 6. Sofosbuvir, Ledipasvir | orgopasvir | (400, 90) mg tablet | Organo Pharma | EGP 1,500 | 28 | 1/2016 |  |
| 7. Sofosbuvir, Ledipasvir | Sofosbuvir + Ledipasvir - Naplex | (400, 90) mg tablet | Naplex pharmaceuticals |  | 28 | 1/2016 |  |
| 8. Sofosbuvir, Ledipasvir | Sofoveravir | (400, 90) mg tablet | AVERROES PHARMA | EGP 1,500 | 28 | 1/2016 |  |
| 9. Sofosbuvir, Ledipasvir | Geneduovir | (400, 90) mg tablet | Genesis Pharmaceuticals | EGP 1,750 | 28 | 2/2016 |  |
| 10. Sofosbuvir, Ledipasvir | Ledisbuvir | (400, 90) mg tablet | IPC Pharma | EGP 1,100 | 28 | 2/2016 |  |
| 11. Sofosbuvir, Ledipasvir | Sofosbuvir & Ledipasvir -Genesis | (400, 90) mg tablet | Genesis healthcare for manufacturing |  | 28 | 4/2016 |  |
| 12. Sofosbuvir, Ledipasvir | Sofocivir Plus | (400, 90) mg tablet | Zeta Pharma |  | 28 | 5/2016 |  |
| 13. Sofosbuvir, Ledipasvir | Virosopasvir | (400, 90) mg tablet | United Company | EGP 1,750 | 28 | 2/2017 |  |
| 14. Sofosbuvir, Ledipasvir | Atcosodivir | (400, 90) mg tablet | ATCO Pharma |  | 28 | 3/2017 |  |
| 15. Sofosbuvir, Ledipasvir | Baxybuvir | (400, 90) mg tablet | Sun Pharma Egypt | EGP 1,100 | 28 | 11/2017 |  |
| 16. Sofosbuvir, Ledipasvir, | MyhepLvir | (400, 90) mg tablet | One Pharma Tech | EGP 1,500 | 28 | 9/2016 |  |
|  |  |  |  |  |  |  |  |
| Sofosbuvir, Ledipasvir, Glecaprevir | Neolipasvir® | (400, 90, 90) mg tablet | RIVA PHARMA | EGP 1,100 | 28 | 3/2016 |  |
|  |  |  |  |  |  |  |  |
| Sofosbuvir, Velpatasvir, | Heterovelpa® | (400, 100, 100) mg tablet | Pharmed health care | EGP 2,000 | 28 | 1/2019 |  |
|  |  |  |  |  |  |  |  |
| Grazoprevir, Elbasvir | Zepatier® | 100, 50 /mg tablet | MERCK SHARP&DOHME (MSD) | EGP 27,000 | 28 | 12/2016 |  |
